# Specific collagen peptides benefit the biosynthesis of matrix molecules of tendons and ligaments

**DOI:** 10.1186/1550-2783-10-S1-P23

**Published:** 2013-12-06

**Authors:** M Schunck, S Oesser

**Affiliations:** 1 CRI, Collagen Research Institute, Kiel, Germany

## Background

An intact composition of extracellular matrix (ECM) collagens, proteoglycans and elastic fibres are responsible for the constitutional strength of tendons and ligaments [1,2]. It is known that pathophysiological changes in the ECM could lead to reduced extension properties and diminished capacity of energy absorption of ligaments and tendons and could promote diseases like patellar tip syndrome, tendinopathy and rupture [3,4].

In a clinical study it could be demonstrated that the oral ingestion of specific collagen peptides improved extension properties of the finger joints [5].

Aim of the present study was to investigate the impact of a specific collagen peptide composition (FORTIGEL^®^) on the extracellular matrix of ligaments and Achilles tendons. Previous experimental studies confirmed the stimulatory impact of these bioactive collagen peptides on the ECM biosynthesis of joint cartilage tissue [6-8].

## Methods

Primary fibroblasts derived from human ligaments and tendons were isolated by enzymatic digestion and seeded in monolayer cultures in a humidified incubator in 5 % CO_2_ atmosphere at 37° C. After 80 % cell confluence regular culture medium was supplemented with 0.5 mg/ml of a specific collagen hydrolysate (FORTIGEL^®^, GELITA AG, Germany).

The RNA expression of matrix molecules and degenerative metalloproteinases was determined via real-time PCR after a stimulation time period of 24 h. Moreover, the collagen, proteoglycan and elastin biosynthesis of tendon and ligament derived fibroblasts was determined using validated methods like western blotting, alcian blue staining or ^14^[C]-incorporation assay.

## Results

The biosynthesis of ligament and tendon matrix molecules was clearly stimulated by FORTIGEL^®^. The RNA expression and biosynthesis of collagen type I and III statistically significantly increased 1.2 fold to 2.4 fold in comparison to untreated control, respectively. In addition, the synthesis of proteoglycans (versican, decorin), was increased in both Achilles tendons and ligament fibroblasts.

Moreover, a statistically significant increase in the elastin biosynthesis, the most prominent component of ligament matrix, was detected. FORTIGEL^®^ treatment leads to an approximately 50 % higher elastin synthesis compared to the untreated control cells.

In contrast to these stimulatory effects the expression of matrix metalloproteinases was down regulated in both tissues after administration of the specific collagen peptides.

## Conclusion

The results indicate that the specific collagen hydrolysate has a pronounced, statistically significant stimulatory impact on the biosynthesis of extracellular matrix molecules in tendons and ligament cells. Although more clinical data are desirable a FORTIGEL^®^ administration seems to be an interesting option for the treatment and prevention of pathological changes in ligaments and tendons like tendinopathy and might reduce the risk of injuries and rupture.
